# Effect of Adsorbent Pretreatments on Methanol Content and Quality of Jujube Wine

**DOI:** 10.3390/foods15071223

**Published:** 2026-04-03

**Authors:** Ang Li, Jianhua Guo, Wan Zhao, Changbao Sun

**Affiliations:** College of Food and Biological Engineering, Qiqihar University, Qiqihar 161006, China; liang621liang@126.com (A.L.); gjh19771123@163.com (J.G.); zhaohan.14@163.com (W.Z.)

**Keywords:** jujube wine, methanol content, adsorbent pretreatment, sensory evaluation

## Abstract

Jujube wine production faces the technical challenge of excessive methanol formation due to the high pectin content of the raw material. This study systematically compared five pre-fermentation treatments (pectinase, chitosan, bentonite, gelatin, and diatomite) for their effects on the methanol content and overall quality of jujube wine, aiming to identify an adsorbent pretreatment strategy that effectively reduces methanol levels while preserving wine quality. Using pectinase treatment as a reference control (representing conventional industrial pectin degradation), the results showed that all adsorbent treatments reduced the pectin content in jujube juice, thereby influencing methanol generation in the fermented wine. Notably, chitosan pretreatment exhibited the most pronounced methanol reduction (113.35 mg/L), which was 59.20% lower than that of the pectinase reference group (277.65 mg/L), and remained far below the methanol limits stipulated by Chinese, EU, and OIV standards (≤400 mg/L). This effectiveness is attributed to the positively charged nature of chitosan, which efficiently removes negatively charged pectin through electrostatic adsorption, thereby blocking methanol formation at the source. Chitosan treatment also resulted in the highest alcohol content (5.82%) and significantly reduced organic acid levels but concurrently led to a slight decrease in some key aroma esters. However, sensory evaluation revealed that jujube wine produced from the chitosan-pretreated juice maintained a harmonious overall taste profile, with an overall score comparable to that of the pectinase reference group. In comparison, bentonite and gelatin showed moderate effectiveness, while diatomite performed poorly. Notably, although pectinase treatment yielded the lowest pectin content, it paradoxically resulted in the highest methanol levels, highlighting the critical mechanism of pectin degradation (rather than removal) promoting methanol formation. In conclusion, chitosan was recommended as an effective pre-fermentation treatment strategy for producing jujube wine with a significantly reduced methanol risk. This study provided a theoretical basis and technical reference for the safe, high-quality industrial production of jujube wine.

## 1. Introduction

Jujube wine is a nutritious fruit wine made from the characteristic resource jujube through fermentation. It is rich in cyclic adenosine monophosphate, phenolic substances, and various amino acids, and has a unique flavor and health value, with broad market prospects [1]. However, the pectin content of jujube is generally high, which is easily decomposed by pectinase produced by yeast or miscellaneous bacteria during the fermentation process, resulting in a large amount of methanol production [2]. In alcoholic beverages, the residual amount of methanol, as a substance with strong toxicity to the human nervous and visual systems [3,4,5], is strictly limited by domestic and foreign regulations. According to international regulations (e.g., EU Regulation 2019/934 and Chinese National Standard GB 2757-2012) [6,7], the methanol limit for fruit distilled spirits is ≤400 mg/L (based on pure alcohol), and fermented fruit wines generally refer to this standard [8]. Therefore, effectively controlling the methanol content is a key technical bottleneck that urgently needs to be solved for the safe production and quality improvement of jujube wine.

Currently, the strategies for reducing methanol content in fruit wine mainly include screening low-pectin raw materials, optimizing fermentation strains and processes, and conducting pre- or post-fermentation treatments. Among these, pretreatment of the raw juice before fermentation to directly reduce pectin—the precursor substance of methanol—is considered a fundamental approach for source control [8,9,10].

Among various pretreatment methods, the use of food-grade adsorbents for juice clarification and degumming has attracted considerable attention due to their simple operation, controllable cost, and suitability for large-scale production [11,12]. Adsorbents selectively remove colloids, proteins, and macromolecular pectin from fruit juice through mechanisms such as electrostatic adsorption, hydrogen bonding, complexation, or physical retention, thereby reducing the amount of substrate available for methanol generation [13,14,15]. In addition, studies have shown that adding adsorbents before fermentation was more beneficial for improving the sensory quality of fruit wine compared to post-fermentation treatments [16,17,18].

Although previous studies have confirmed the effectiveness of adsorption pretreatment for clarifying fruit wines and mitigating methanol, a systematic comparative investigation specifically targeting jujube wine as a matrix is still lacking. Existing research has largely focused on the effect of a single adsorbent on a single indicator (such as methanol content or clarity). However, the comparative efficiency of different adsorbents in removing pectin from jujube juice, and their comprehensive impact on subsequent methanol generation, dynamic changes in physicochemical indicators, organic acid profiles, aroma characteristics, and sensory quality during fermentation have not been systematically reported. Furthermore, due to their different mechanisms of action, various adsorbent types (e.g., chitosan, bentonite, gelatin, and diatomaceous earth) may differentially affect the reducing sugars, acids, and flavor precursors in jujube juice, thereby significantly altering the fermentation process and the final wine flavor. This critical scientific question remains underexplored.

Therefore, this study focused on fresh jujube juice and employed four common food-grade adsorbents (chitosan, bentonite, gelatin, and diatomaceous earth) for pretreatment before fermentation, with pectinase treatment serving as a reference control. The objectives were to: (1) compare the pectin removal efficiency of different adsorbents from jujube juice and their inhibitory effects on the methanol content in the fermented wine; (2) dynamically monitor the impact of adsorption pretreatment on key physicochemical indicators (soluble solids, total reducing sugar, total acidity, and pH) during fermentation; and (3) comprehensively evaluate the effects of different treatments on the overall quality of jujube wine through organic acid profile analysis, electronic nose aroma fingerprinting, and sensory evaluation. Through this multi-dimensional systematic comparison, this study aims to identify an effective adsorbent pretreatment strategy that could efficiently reduce the methanol content while preserving the physicochemical properties and sensory flavor of jujube wine to the greatest extent possible, thereby providing a theoretical basis and technical reference for the standardized, safe, and high-quality production of jujube wine.

## 2. Materials and Methods

### 2.1. Materials

Dried and undamaged jujubes were purchased from Aksu, Xinjiang Uyghur Autonomous Region, China. Pectinase (activated, 60,000 U/g) was procured from LAFFORT (Bordeaux, France). *Saccharomyces cerevisiae BV818* was purchased from Angel Yeast Co., Ltd. (Yichang, China). Chitosan (food-grade, degree of deacetylation ≥ 90%), bentonite (sodium bentonite, food-grade), gelatin (type A, derived from porcine skin, food-grade), and diatomite (calcined, food-grade) were purchased from Xintai Industrial Co., Ltd. (Shanghai, China). All other chemical reagents were of at least analytical grade.

### 2.2. Jujube Wine Making

**Figure 1:**
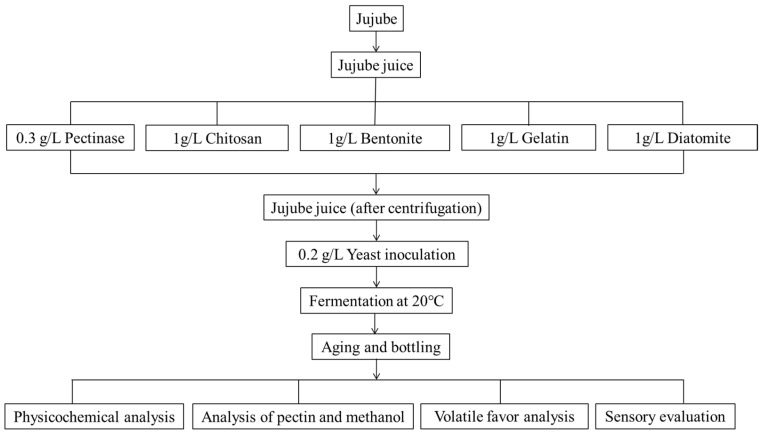
Schematic diagram of jujube wine-making process.

The jujube wine was prepared according to previously described methods [10] with slight modifications. Dried, undamaged jujubes were selected, washed, and rehydrated at a ratio of 1:8 (jujube:water) in a thermostat water bath at 80 °C for 24 h. The rehydrated jujubes were manually destoned and crushed using a laboratory juicer (Qingdao China-Belgian Brewing Co., Ltd., Qingdao, China). Jujube juice was collected and divided into five groups for the different pre-fermentation treatments. The pectinase group was defined as the reference control as it represents the conventional industrial practice for pectin degradation through enzymatic hydrolysis rather than adsorption. For this group, pectinase was added at a concentration of 0.3 g/L and incubated for 150 min. The four experimental groups were treated with food-grade adsorbents: chitosan, bentonite, gelatin, and diatomite, each added at a concentration of 1 g/L and allowed to stand for 24 h. After the respective treatments, all juices were centrifuged (Suzhou Degao Centrifuge Manufacturing Co., Ltd., Suzhou, China) to remove the added agents. The clarified juices were then inoculated with 0.2 g/L active dry yeast (*Saccharomyces cerevisiae BV818*, Angel Yeast Co., Ltd., China, rehydrated before inoculation, with an initial viable cell count of approximately 10^7^ CFU/mL) and fermented at 20 °C. Fermentation was monitored daily by measuring the total reducing sugar content and was considered complete when no further decrease in total reducing sugar content was observed for seven consecutive days. The resulting young wines were transferred to new vessels and aged at 5 °C for 25 days. All experiments were performed in triplicate independent fermentation batches (biological replicates), with three technical replicates per sample. The physicochemical indices of all groups were quantified daily during fermentation. A schematic diagram of the jujube wine-making process is presented in Figure 1.

### 2.3. Determination of Main Physicochemical Indicators

The total reducing sugar content (expressed as glucose, g/L), total acidity (g/L), soluble solid content (g/L), and dry extract content (g/L) were measured in accordance with the Chinese National Standard GB/T 15038-2006 (General Analysis Method for Wine and Fruit Wine) [19]. The pH value was measured using a digital pH meter (HI99163, Hanna Instruments, Shanghai, China). The pectin content was determined as described in Section 2.5.

### 2.4. Determination of Organic Acid Content

Organic acids in jujube wine were measured by high-performance liquid chromatography (Agilent 1260 Infinity II, Agilent Technologies, Santa Clara, CA, USA).

Chromatographic Conditions: An Agilent 1260 Infinity II HPLC system equipped with a Zorbax Eclipse Plus C18 column (250 mm × 4.6 mm, 5 μm) was used. The mobile phase was a 0.02 mol/L potassium dihydrogen phosphate solution (pH 2.8) at a flow rate of 0.8 mL/min. Isocratic elution was performed at 30 °C with an injection volume of 20.0 μL. The detection wavelength was set at 210 nm.

Sample Preparation: A 5.0 mL aliquot of jujube wine was centrifuged at 10,000 rpm for 10 min. The supernatant was filtered through a 0.22 μm membrane filter prior to HPLC analysis.

Qualitative and Quantitative Analysis: Organic acids were identified by comparing retention times with authentic standards and confirmed by standard addition amounts. Quantification was performed using the external standard method. Calibration curves were constructed by plotting peak areas against standard concentrations, and sample concentrations were calculated accordingly. Each sample was analyzed in triplicate.

Method Validation: The method showed good linearity with correlation coefficients (R^2^) > 0.999 for all organic acids. Recovery ranged from 92.30% to 105.70%, with acceptable precision.

### 2.5. Analysis of Pectin

The pectin in jujube juice was obtained after adsorbent treatment according to the methods of the International Federation of Fruit Juice Producers (IFU, 2000) [20].

#### 2.5.1. Extraction of Pectin

After adding 35.0 mL of absolute ethyl alcohol preheated to 75 °C into 15.0 mL of the jujube wine samples, the mixed liquid was placed into a thermostat water bath at 85 °C, and then thoroughly stirred for 10 min. The total volume was brought to 50.0 mL by adding absolute ethyl alcohol (85 °C), and the resulting mixture was centrifuged at 4000× *g* for 15 min to remove the supernatant. The sediment was washed with 5.0 mL of 67% ethanol (85 °C) in a water bath at 85 °C for 10 min. Then, it was centrifuged at 4000× *g* for 15 min to remove the supernatant. This step was repeated until there was no Molisch’s reaction (after adding α-naphthol and sulfuric acid, no purple color ring appeared at the interface between the two liquid layers) in the supernatant. The residue obtained was dissolved in deionized water for subsequent analysis.

#### 2.5.2. Preparation of Galacturonic Acid Standard Curve

Pectin generates galacturonic acid after hydrolysis, which undergoes a condensation reaction with carbazole, resulting in a purple-red compound. This compound has a maximum absorption peak at 525 nm, and its absorption value was proportional to the content of pectin. The standard curve was established by using a series of galacturonic acid solutions with concentrations of 0 mg/L, 10.0 mg/L, 20.0 mg/L, 40.0 mg/L, 60.0 mg/L, and 100.0 mg/L. The absorbance was then recorded at 525 nm using a UV–VIS spectrophotometer (Unico 2600A, Shanghai, China) to plot the standard curve with the corresponding absorbance value as the ordinate and the galacturonic acid content as the abscissa.

#### 2.5.3. Quantification of Galacturonic Acid Content in Jujube Wine Samples

The method of quantifying the galacturonic acid content in the jujube wine samples was the same as the method for generating the galacturonic acid standard curve mentioned above. According to the standard curve, the galacturonic acid and pectin contents of the jujube wine samples were calculated.

### 2.6. Quantification of Alcohol and Methanol

The alcohol (% by volume) and methanol contents were determined using a gas chromatography system (GC-2010 Plus, Shimadzu, Kyoto, Japan) by adding an internal standard according to the reference method of AOAC Official Methods 972.11 and 983.13 (AOAC, 2019) [8,21,22]. The detailed parameters for methanol determination were as follows: calibration range of 10–500 mg/L (R^2^ = 0.999), detection limit of 3 mg/L, quantification limit of 10 mg/L, spike recovery of 95–105%, and intra- and inter-day precision RSD < 3%. A 50.0 mL volume of deionized water was added to 100.0 mL of a jujube wine sample and distilled to a volume of 100.0 mL. A 10.0 mL volume of the distillate and one milliliter of internal standard (162.0 mg/L tert-amyl alcohol) were mixed, and then 1.0 μL of the composite sample was directly injected into the GC system equipped with a flame ionization detector and a PEG-20 M capillary column (30 m × 0.5 mm × 0.25 μm, Hangzhou FPI Scientific Instrument, Hangzhou, China). The temperature of the detector and injector was 220 °C. The oven temperature was held at 40 °C for 5 min, increased to 200 °C at a rate of 4 °C/min, and then immediately increased to 220 °C at 10 °C/min and held for 10 min. Nitrogen gas was used as the carrier gas at a flow rate of 1.0 mL/min; the split ratio was 50:1.

### 2.7. Volatile Aroma-Active Component Analysis by HS-SPME-GC-MS

An 7890A-5975C gas chromatography–mass spectrometer (GC-MS) equipped with an HP-5MS capillary column (30 m × 250 μm × 0.25 μm; Agilent Technologies, Santa Clara, CA, USA) was used for qualitative and quantitative analyses of the volatile compounds in jujube wine.

Sample Preparation: A 5.0 mL wine sample was placed in a 15.0 mL headspace vial, and 1 g of NaCl was added. The vial was immediately sealed with a PTFE–silicone septum. The sample was equilibrated at 50 °C for 20 min, and then an SPME fiber (50/30 μm DVB/CAR/PDMS, Supelco, Bellefonte, PA, USA) was inserted into the headspace vial for extraction at 50 °C for 30 min. After extraction, the fiber was inserted into the gas chromatograph inlet and desorbed at 250 °C for 3 min.

Chromatographic Conditions: An HP-5MS capillary column (30 m × 250 μm × 0.25 μm, Agilent Technologies, USA) was used. The temperature program was as follows: initial temperature of 60 °C, which was held for 2 min, then increased to 110 °C at a rate of 5 °C/min and held for 2 min, then increased to 220 °C at 10 °C/min and held for 10 min. High-purity helium (≥99.999%) was used as the carrier gas at a flow rate of 1.0 mL/min in splitless mode. The interface temperature was set at 230 °C.

Mass Spectrometry Conditions: A 5975C quadrupole mass spectrometer (Agilent Technologies, USA) was used with an electron ionization source at 70 eV. The ion source temperature was 230 °C, the quadrupole temperature was 150 °C, and the mass scan range was 45–450 amu.

Qualitative and Quantitative Analyses: Volatile compounds were identified by comparison with the NIST 17 mass spectral library, combined with retention indices (calculated using C7–C40 n-alkane standards) and comparison with literature values for confirmation. Quantitative analysis was performed using the peak area normalization method to calculate the relative percentage content of each volatile compound. Each sample was analyzed in triplicate.

### 2.8. Sensory Evaluation

The sensory evaluation was conducted according to Chira and Teissedre [23]. A panel of 10 trained professional tasters (5 females and 5 males, all with experience in fruit wine evaluation) scored the samples using a 10-point scale (1 = extremely poor, 10 = excellent). All samples were presented with three-digit random codes, and tasters evaluated them in individual booths. The mean values were used for statistical analysis.

### 2.9. Statistical Analysis

In the present study, statistical analysis was performed using SPSS 22.0 (SPSS-IBM Inc., Chicago, IL, USA). One-way analysis of variance (ANOVA) was applied to analyze the difference in the results. Mean differences with *p* < 0.05 according to the Tukey test were considered significant. All data are the average values of three biological replicates, which were analyzed in triplicate for each studied condition, and are presented as the mean ± deviation.

## 3. Results and Discussion

### 3.1. Effect of Different Adsorbents on the Physicochemical Indices of Jujube Juice

As shown in Table 1, all adsorbent treatments did not result in significant changes in the total reducing sugar content, total acidity, or pH of jujube juice (*p* > 0.05), but all adsorbents significantly reduced the soluble solid and pectin contents. The chitosan group had the lowest soluble solid (90.41 g/L) and pectin contents (66.19 mg/L), the bentonite group’s results were close to those of the gelatin group, and the diatomite group’s results were close to those of the untreated juice, which stemmed from the amino groups of the chitosan molecule chains carrying a large amount of positive charge after protonation in acidic juice [24,25]. These chitosan molecules with positive charges had a strong adsorption ability for negatively charged colloidal substances (pectin, proteins, polyphenol polymers, etc.), which disrupted the stability of the colloid, which caused the colloid to aggregate, settle, and be removed. Bentonite had a strong adsorption capacity and removed pectin after adsorbing it. Bentonite adsorbs pectin through hydrogen bonding and electrostatic interactions with its layered silicate structure. The polyphenolic molecule linked with the amino groups (-NH_2_) and the non-polar amino acid side chains of the gelatin molecule chains through hydrogen bond formation and hydrophobic interactions, forming a loose flocculent substance that was precipitated [26,27]. In contrast, diatomite had the lowest adsorption capacity among these adsorbents. Diatomite mainly removed large particles through physical entrapment, but its surface charge was weak, limiting its adsorption capacity for pectin, resulting in the poorest effect.

### 3.2. Effect of Different Adsorbents on the Physicochemical Indices of Jujube Wine

As shown in Table 2, the chitosan group exhibited the lowest total acidity content (6.29 g/L) and total reducing sugar content (1.10 g/L) among all the treatments. Simultaneously, the chitosan group showed a significantly higher pH value (4.94) and alcohol content (5.82%) compared to the pectinase reference group and the other experimental groups (*p* < 0.05). Although the pH changes were numerically small (0.1–0.2 units), they have biologically significant effects on microbial growth and enzyme activity, particularly pectinase and esterase activity, indirectly influencing methanol and aroma compound formation. The reduction in buffering capacity of the jujube wine fermented from chitosan-pretreated juice could be attributed to the strong adsorption capacity of chitosan for buffering substances, particularly organic acids [26,28]. This interpretation was supported by the significantly lower dry extract content in the chitosan group compared to the pectinase group and the other experimental groups. The removal of pectin by chitosan also likely altered the fermentation dynamics. Pectin, as a structural polysaccharide, could affect yeast cell wall integrity and metabolic activity. The reduction in pectin content in the fermentation medium might have enhanced yeast access to simple sugars, potentially explaining the higher alcohol yield in the chitosan group [29]. Furthermore, the altered acid profile might have shifted the metabolic balance of yeast toward alcohol production rather than organic acid synthesis, contributing to both the reduced acidity and increased ethanol content.

### 3.3. Effect of Different Adsorbents on the Organic Acid Content of Jujube Wine

Organic acids directly affect the total acidity, pH, flavor, taste, and even the stability of the wine body [29]. As shown in Table 3, the organic acids in jujube wine mainly include malic acid, succinic acid, lactic acid, acetic acid, citric acid, pyruvic acid, and fumaric acid. The jujube wine fermented from the chitosan-treated juice showed significant decreases (*p* < 0.05) in malic acid, acetic acid, succinic acid, pyruvic acid, and total organic acid contents, consistent with the reduced total acidity observed in this group. This reduction could be explained by the protonation of chitosan molecules due to the acidic environment, forming -NH^3+^ groups that combined with the dissociated organic acid anions through electrostatic adsorption, thereby reducing the organic acid content [30,31]. The removal of organic acids by chitosan has important implications for wine flavor beyond simple acidity reduction. Organic acids serve as precursors for ester formation during fermentation and aging; therefore, their reduction may influence the ester profile of the final wine. The balance between acids and esters is critical for sensory perception, as acids provide freshness and structure, while esters contribute fruity and floral notes. The jujube wine fermented from the bentonite-treated juice also had a reduced organic acid content, though it was less pronounced than the chitosan group. This was probably due to the hydrogen-bond interactions between -SiO (-AlO) groups of bentonite and the -OH of organic acid molecules [32]. Gelatin, which carries a positive charge under acidic conditions, interacted with negatively charged organic acid anions through electrostatic attraction, indirectly reducing the organic acid and pectin contents. In contrast, the diatomite group was not significantly different from the pectinase group (*p* > 0.05), reflecting its limited adsorption capacity for charged molecules.

### 3.4. Effect of Different Adsorbents on the Content of Pectin and Methanol of Jujube Wine

**Figure 2:**
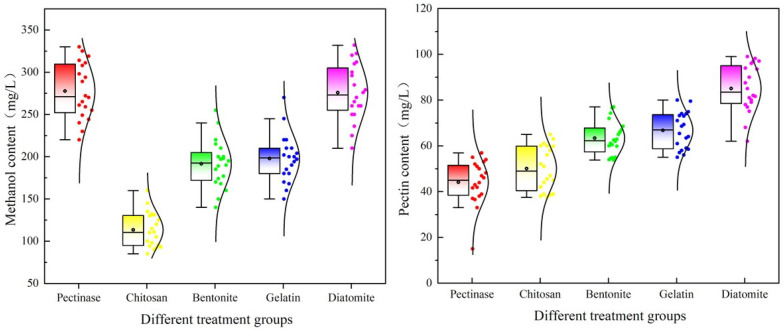
Effects of different treatments on pectin and methanol contents in jujube wine.

As shown in Figure 2, the pectin and methanol contents varied considerably among the treatments. The pectinase group exhibited the lowest pectin content (44.09 mg/L) but the highest methanol content (277.65 mg/L). This paradox arises from the enzymatic mechanism: pectinase hydrolyzes the methyl ester bonds of galacturonic acid in pectin, releasing methanol as a byproduct [5]. Thus, while pectinase effectively reduces pectin (44.09 mg/L), it simultaneously generates methanol (277.65 mg/L). In contrast, the chitosan group has a slightly higher residual pectin content (50.07 mg/L) but achieved the lowest methanol content (113.35 mg/L), representing a 59.20% reduction compared to the pectinase group. This remarkable methanol reduction was attributed to chitosan’s efficient adsorption of negatively charged pectin and colloidal substances through electrostatic interactions [33]. By removing pectin rather than degrading it, chitosan prevents the enzymatic release of methanol during fermentation. The methanol content in the chitosan group (113.35 mg/L) was far below the safety limit (Chinese and EU: ≤400 mg/L; OIV: ≤300 mg/L), while the pectinase group (277.65 mg/L), although compliant, approached the OIV limit. The bentonite and gelatin groups showed intermediate results, with higher pectin contents (63.37 mg/L and 66.75 mg/L) and methanol contents (191.65 mg/L and 197.75 mg/L). Gelatin carries a positive charge under acidic conditions and removed pectin through electrostatic attraction and flocculation. The diatomite group showed the poorest adsorption capacity (pectin: 85.03 mg/L; methanol: 275.91 mg/L), with no significant difference from the pectinase group (*p* > 0.05), reflecting its predominantly physical retention mechanism with limited surface charges. The removal of pectin by adsorbents also influenced fermentation metabolism. Pectin can bind to yeast cell surfaces, potentially affecting nutrient uptake and metabolic efficiency. By removing pectin, chitosan may enhance yeast access to fermentable sugars, contributing to the higher alcohol content observed in this group. Furthermore, the altered matrix composition might shift yeast metabolic pathways, influencing the balance between primary (ethanol) and secondary (methanol, higher alcohols, and esters) metabolites. The reason could be that gelatin carries a positive charge in acidic conditions and forms flocs with negatively charged pectin through electrostatic attraction, thereby removing pectin [33].

### 3.5. Effect of Adsorbents on the Volatile Aroma-Active Compounds in Jujube Wine

**Figure 3:**
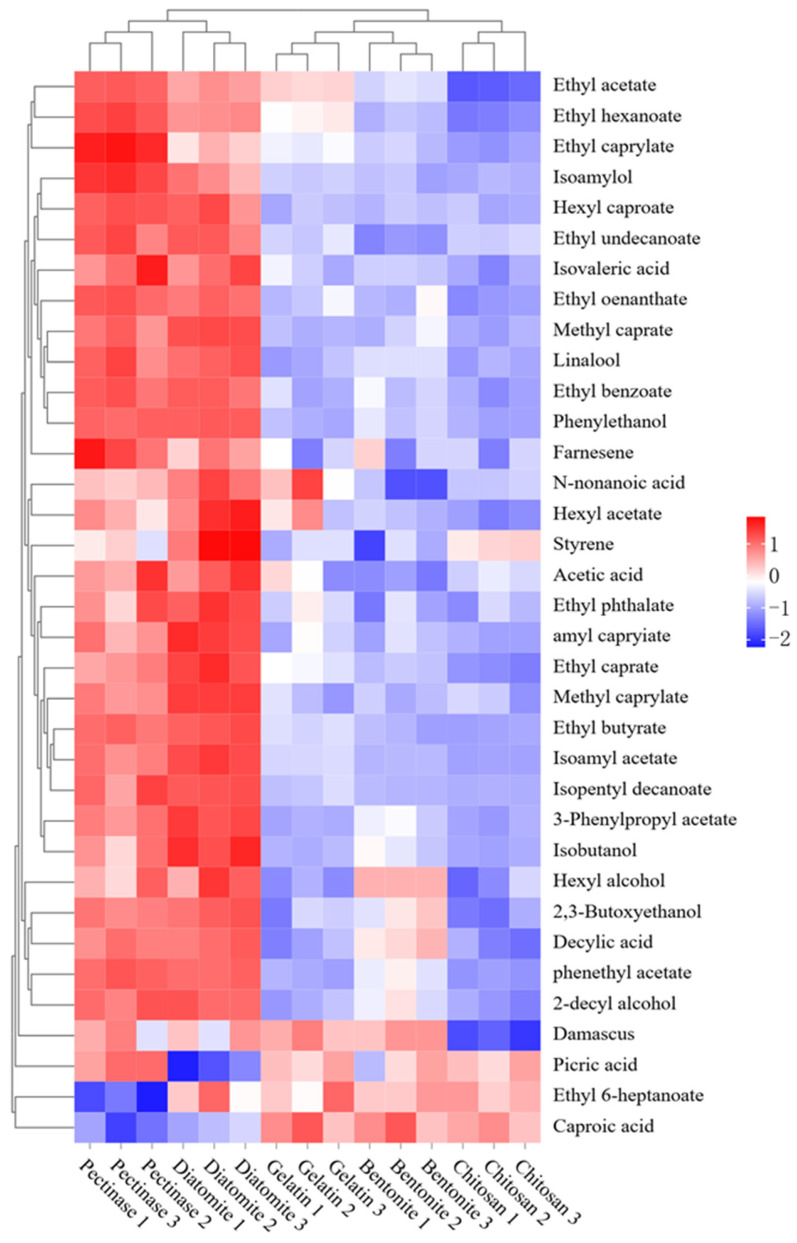
Hierarchical cluster analysis of volatile aroma-active components in jujube wine.

**Figure 4:**
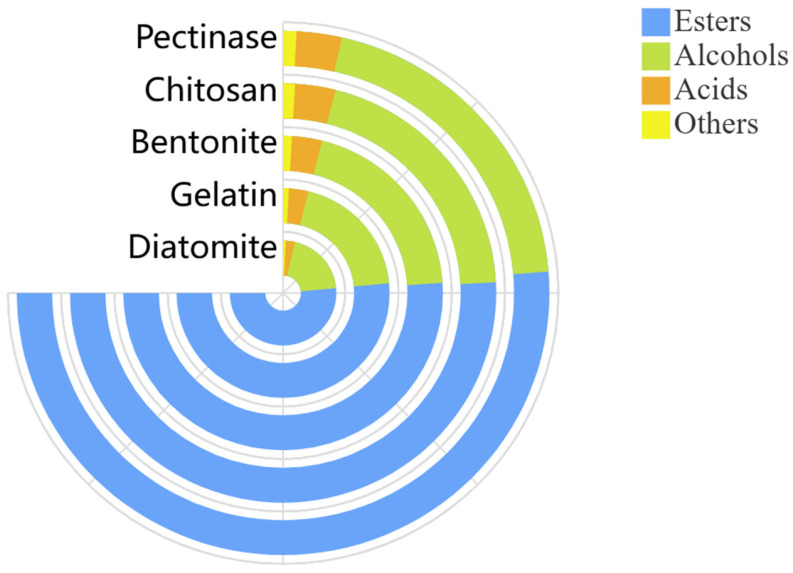
Ring chart analysis of relative percentage contents of volatile aroma-active components in jujube wine. Ring chart reflects numerical values through angles, not lengths.

When investigating the effect of different treatments on the aroma-active components of jujube wine, hierarchical cluster analysis could be a useful tool. The results are shown as a color range from red to blue, which indicate high to low contents. HS-SPME-GC-MS analysis identified a total of 35 volatile compounds across all samples, including 19 esters, seven alcohols, six acids, and three other components (Figure 3). The chitosan group showed a slightly lower total ester content (approximately 67.76%) compared to the pectinase group (approximately 68.65%), consistent with the reduced organic acid precursors available for esterification (Figure 4). The relationship between organic acids and esters is critical for understanding the flavor impact of adsorbent treatments. Esters are primarily formed through two pathways during fermentation: (1) enzymatic esterification catalyzed by yeast alcohol acetyltransferases using higher alcohols and acetyl-CoA as substrates, and (2) acid-catalyzed esterification between organic acids and alcohols during aging. The reduction in organic acids by the chitosan treatment directly limits substrate availability for the latter pathway, partially explaining the reduced ester content. However, the sensory significance of this reduction should be considered in context. Higher alcohols, which were also slightly reduced in the chitosan group, have high sensory thresholds and thus contribute minimally to overall aroma perception [34]. In contrast, esters such as ethyl hexanoate (pineapple aroma), ethyl decanoate (pear aroma), and ethyl octanoate (apple aroma) had low sensory thresholds and are key contributors to fruity notes. The moderate reduction in these esters in the chitosan group was not sufficient to negatively impact overall sensory scores, suggesting that the remaining ester concentration remained above perception thresholds. Notably, the chitosan group retained the characteristic ester profile of jujube wine while achieving a significant methanol reduction. This indicated that chitosan selectively adsorbed pectin and organic acids without completely stripping the wine of its aromatic potential. The synergistic interactions among the remaining esters, alcohols, and other volatile compounds likely contributed to the harmonious sensory profile observed in the sensory evaluation [35]. The diatomite group showed ester profiles similar to the pectinase group, consistent with its poor pectin adsorption capacity. The bentonite and gelatin groups exhibited intermediate ester profiles, reflecting their moderate removal of pectin and organic acid precursors.

### 3.6. Sensory Evaluation

This part explored the effects of the different adsorbents on four aspects of jujube wine, including appearance, odor, taste, and texture. Statistical analysis was performed for each sensory attribute, with significant differences indicated in the radar chart.

**Figure 5:**
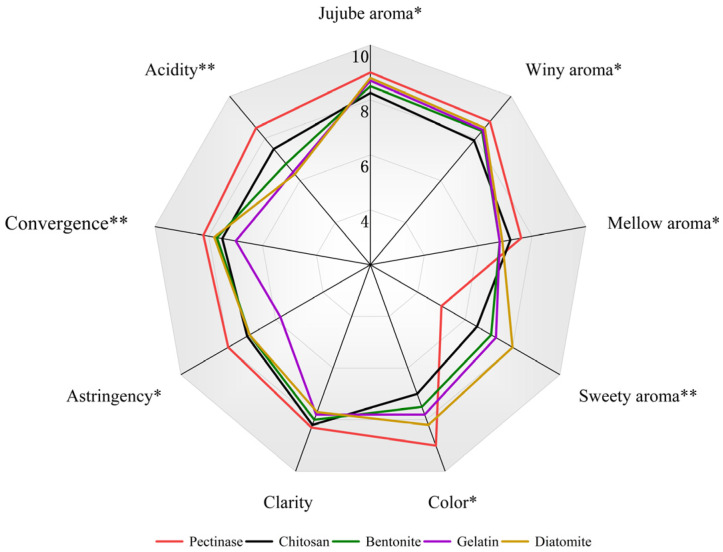
Radar graph of the mean sensory scores of jujube wine. Note: * indicates significance at *p* < 0.05; ** indicates significance at *p* < 0.01.

As shown in Figure 5, the different adsorbent pretreatments significantly affected the sensory quality of jujube wine. The radar chart revealed that all treatment groups produced clear, transparent wines with a light jujube color. Notably, the color scores of the gelatin and diatomite groups were significantly higher than those of the chitosan group (*p* < 0.05), indicating that the chitosan treatment resulted in a lighter wine color. Regarding aroma, the pectinase group (reference control) received the highest scores for jujube aroma, winy aroma, and mellow aroma. Compared to the pectinase group, the chitosan group showed significantly lower scores for these aroma attributes (*p* < 0.05), consistent with the reduced ester compound content in the chitosan group reported in Section 3.5. The bentonite and gelatin groups exhibited intermediate aroma scores between the chitosan and pectinase groups, while the diatomite group showed no significant differences from the pectinase group (*p* > 0.05). In terms of taste, all adsorbent treatments significantly reduced astringency, convergence, and acidity (*p* < 0.05), which was primarily attributed to the decreased polyphenol and organic acid contents. The gelatin group received the lowest scores for astringency, convergence, and acidity, but this also resulted in a bland and less layered wine. The chitosan group, while showing reduced scores for these taste attributes, maintained good overall harmony, with moderate acidity and no obvious bitterness or astringency. Regarding texture, all treatment groups received high scores (>7.5), with no significant differences between the groups (*p* > 0.05), indicating that all jujube wines had good mouthfeel and smoothness. The overall scores (average of the four attributes) showed that the pectinase group (8.0) and chitosan group (7.9) received the highest scores, with no significant difference between them (*p* > 0.05). The bentonite (7.5) and gelatin (7.3) groups followed, while the diatomite group (7.1) received the lowest overall score. Although the chitosan treatment led to a reduction in some aroma compounds, the overall sensory quality remained well-balanced, with an overall score comparable to that of the pectinase group.

### 3.7. Effect of Adsorbents on the Quality of Jujube Wine

In investigating the effect of the different adsorbent pretreatments on the quality of jujube wine, hierarchical cluster analysis can be a useful tool. Hierarchical cluster analysis was performed on the differences in alcohol content, total reducing sugar content, total acidity, pH, dry extract content, and malic acid, lactic acid, succinic acid, acetic acid, pyruvic acid, citric acid, fumaric acid, total organic acids, pectin, and methanol contents in jujube wine. The results are shown as a color range from blue to red, indicating high to low contents.

**Figure 6:**
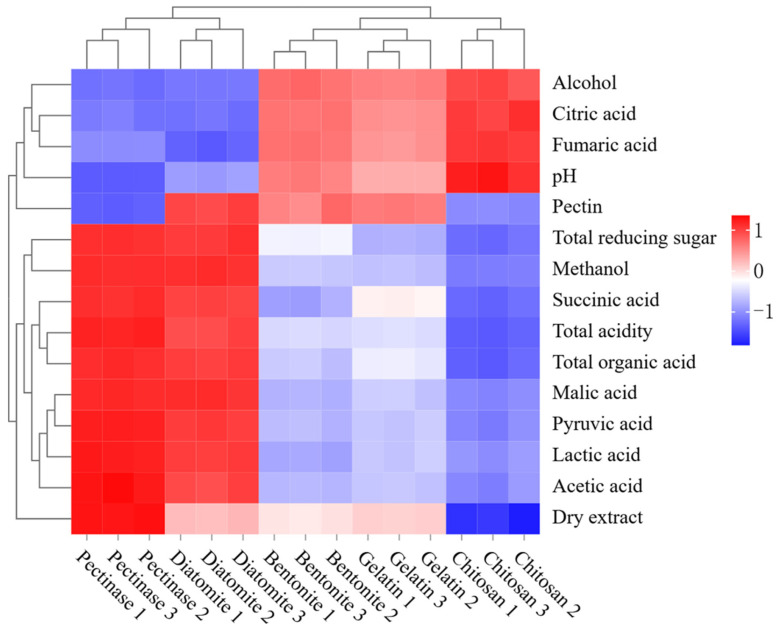
Hierarchical cluster analysis of jujube wine.

As shown in Figure 6, the total acidity, total organic acid content, dry extract content, and total reducing sugar content in the chitosan group were significantly decreased, while the pH and alcohol content were significantly enhanced. Meanwhile, the pectin and methanol contents were significantly lower than in other groups. Bentonite and gelatin had a similar effect on jujube wine. Compared with chitosan, the jujube wine treated with bentonite and gelatin had higher methanol contents. Among all the samples, the diatomite group had the weakest adsorption capacity for pectin and the highest methanol content, which was close to that of the pectinase group.

### 3.8. Statement of Limitations

While this study provided valuable insights, several limitations should be acknowledged: (1) This study used the pectinase group as a reference control but did not include an untreated jujube juice blank control as untreated juice presents fermentation difficulties and is rarely used in industrial practice. However, the lack of a blank control limits a comprehensive assessment of the absolute effectiveness of the adsorbents. Future studies should include an untreated group to more accurately quantify the net effects. (2) The sensory evaluation was conducted by a panel of 10 trained tasters, all experienced in fruit wine evaluation, with replicate measurements and random coding to ensure reliability. However, the relatively small sample size may result in insufficient statistical power. Future research should expand the panel size to obtain more representative sensory data.

## 4. Conclusions

This study systematically evaluated the effects of five pre-fermentation treatments on the methanol content and overall quality of jujube wine. The results demonstrated that pre-fermentation adsorbent treatment is an effective strategy for controlling methanol generation in jujube wine by reducing the pectin content.

Among the tested adsorbents, chitosan exhibited the most pronounced methanol reduction (113.35 mg/L), which was 59.20% lower than that of the pectinase reference group (277.65 mg/L). This effectiveness was attributed to the positively charged nature of chitosan, which efficiently adsorbs negatively charged pectin and colloidal substances. Chitosan treatment also resulted in the highest alcohol content and significantly reduced organic acid levels, but concurrently led to a slight decrease in some key aroma esters. However, sensory evaluation revealed that the jujube wine produced from the chitosan-pretreated juice maintained a harmonious overall taste profile, with an overall score comparable to that of the pectinase group.

In comparison, bentonite and gelatin showed moderate effectiveness, while diatomite performed poorly. Notably, although the pectinase treatment yielded the lowest pectin content, it paradoxically resulted in the highest methanol levels, highlighting the critical mechanism of pectin degradation (rather than removal) promoting methanol formation. Considering the comprehensive evaluation of methanol reduction efficiency, physicochemical changes, and sensory quality, chitosan is recommended as an effective pre-fermentation treatment strategy for producing jujube wine with a significantly reduced methanol risk.

## Figures and Tables

**Table 1:** Physicochemical indices of jujube juice.

Physicochemical Index	Groups				
	Pectinase	Chitosan	Bentonite	Gelatin	Diatomite
Total reducing sugar (g/L)	81.40 ± 0.01 ^a^	80.90 ± 0.01 ^a^	81.20 ± 0.01 ^a^	81.30 ± 0.01 ^a^	81.40 ± 0.01 ^a^
Soluble solids (g/L)	98.80 ± 0.01 ^a^	90.41 ± 0.01 ^b^	92.81 ± 0.02 ^c^	94.83 ± 0.01 ^d^	97.24 ± 0.01 ^a^
Total acidity (g/L)	5.43 ± 0.02 ^a^	5.32 ± 0.01 ^b^	5.37 ± 0.01 ^b^	5.39 ± 0.01 ^a^	5.41 ± 0.01 ^a^
pH	4.71 ± 0.01 ^a^	4.54 ± 0.01 ^b^	4.65 ± 0.01 ^a^	4.66 ± 0.01 ^a^	4.70 ± 0.01 ^a^
Pectin (mg/L)	90.42 ± 0.01 ^a^	66.19 ± 0.01 ^b^	73.54 ± 0.01 ^c^	75.61 ± 0.01 ^d^	88.90 ± 0.01 ^a^

Note: All values are expressed as means ± standard deviation (*n* = 3); different lowercase letters in the same row indicated significant difference (*p* <0.05).

**Table 2:** Physicochemical indices of jujube wine.

Physicochemical Index	Groups				
	Pectinase	Chitosan	Bentonite	Gelatin	Diatomite
Alcohol (%Vol)	5.51 ± 0.01 ^a^	5.82 ± 0.02 ^b^	5.58 ± 0.01 ^a^	5.54 ± 0.02 ^a^	5.52 ± 0.01 ^a^
Total reducing sugar (g/L)	1.60 ± 0.01 ^a^	1.10 ± 0.02 ^b^	1.40 ± 0.01 ^c^	1.30 ± 0.03 ^d^	1.60 ± 0.01 ^a^
Total acidity (g/L)	7.17 ± 0.02 ^a^	6.29 ± 0.03 ^b^	6.89 ± 0.02 ^c^	6.90 ± 0.03 ^d^	7.06 ± 0.01 ^a^
pH	4.80 ± 0.01 ^a^	4.94 ± 0.01 ^b^	4.86 ± 0.02 ^c^	4.84 ± 0.01 ^c^	4.82 ± 0.01 ^a^
Dry extract (g/L)	24.50 ± 0.02 ^a^	21.30 ± 0.03 ^b^	23.30 ± 0.02 ^c^	23.40 ± 0.01 ^c^	24.10 ± 0.02 ^a^

Note: All values are expressed as means ± standard deviations (*n* = 3); different lowercase letters in the same row indicate significant differences (*p* < 0.05).

**Table 3:** Organic acids in jujube wine.

Treatment	Malic Acid (mg/L)	Lactic Acid (mg/L)	Succinic Acid (mg/L)	Acetic Acid (mg/L)	Pyruvic Acid (mg/L)	Citric Acid (mg/L)	Fumaric Acid (mg/L)	Total Organic Acids (g/L)
Pectinase	5967 ± 11 ^a^	202.6 ± 1.2 ^a^	1787 ± 9 ^a^	113.2 ± 1.6 ^a^	14.93 ± 0.99 ^a^	78.6 ± 2.1 ^a^	0.59 ± 0.32 ^a^	8.16 ± 0.02
Chitosan	5278 ± 13 ^b^	99.4 ± 2.0 ^b^	1219 ± 11 ^b^	69.6 ± 1.6 ^b^	8.94 ± 0.67 ^b^	121.6 ± 2.9 ^b^	3.96 ± 0.27 ^b^	6.80 ± 0.03
Bentonite	5649 ± 12 ^c^	115.0 ± 1.7 ^c^	1587 ± 12 ^c^	104.8 ± 1.3 ^c^	12.11 ± 0.32 ^c^	89.8 ± 2.7 ^c^	3.89 ± 0.44 ^b^	7.56 ± 0.03
Gelatin	5719 ± 13 ^d^	120.3 ± 1.5 ^d^	1622 ± 12 ^c^	107.6 ± 1.8 ^c^	12.76 ± 0.48 ^c^	82.2 ± 2.4 ^d^	3.15 ± 0.63 ^c^	7.67 ± 0.03
Diatomite	5907 ± 12 ^a^	199.9 ± 1.7 ^a^	1751 ± 10 ^a^	113.0 ± 1.3 ^a^	14.90 ± 0.65 ^a^	79.1 ± 3.2 ^a^	1.62 ± 0.51 ^d^	8.07 ± 0.03

Note: All values are expressed as means ± standard deviations (*n* = 3); different lowercase letters in the same column indicate significant differences (*p* < 0.05).

## Data Availability

The original contributions presented in this study are included in the article. Further inquiries can be directed to the corresponding author.
